# Anti-tumour necrosis factor alpha therapy improves insulin sensitivity in normal-weight but not in obese patients with rheumatoid arthritis

**DOI:** 10.1186/ar3900

**Published:** 2012-07-05

**Authors:** Antonios Stavropoulos-Kalinoglou, Giorgos S Metsios, Vasileios F Panoulas, Peter Nightingale, Yiannis Koutedakis, George D Kitas

**Affiliations:** 1 Department of Sport and Exercise Science, University of Thessaly, Trikala- Karyes Road, Trikala, 42100, Greece; 2 Institute of Human Performance and Rehabilitation, Centre for Research and Technology, Trikala, 42100, Greece; 3 Department of Rheumatology, Dudley Group NHS Foundation Trust, Russell's Hall Hospital, Pensnett Road, Dudley, DY1 2HQ, West Midlands, UK; 4 School of Sport, Performing Arts & Leisure, Wolverhampton University, Gorway Road, Walsall, WS1 3BD, West Midlands, UK; 5 Wolfson Laboratory, Department of Medical Statistics, School of Medicine, University of Birmingham, Queen Elizabeth Medical Centre, Edgbaston, Birmingham, B15 2TH, UK; 6 Arthritis Research UK Epidemiology Unit, University of Manchester, Oxford Road, Manchester, M13 9PT, UK

## Abstract

**Introduction:**

Insulin resistance (IR), a risk factor for the development of cardiovascular disease, is common among patients with rheumatoid arthritis (RA). Inflammation, and especially tumour necrosis factor alpha (TNFα), has been associated with IR, and the administration of anti-TNFα agents is suggested to improve insulin sensitivity. However obesity, a potent contributor to IR, may limit the beneficial effects of anti-TNFα medication on IR. The aim of this study is to compare the effects of anti-TNFα therapy on IR between normal-weight and obese patients with RA.

**Methods:**

Patients who were normal-weight with IR (N+IR) or obese with IR (O+IR) and had embarked on anti-TNFα treatment, participated. Assessments included body mass index (BMI), insulin sensitivity (Homeostasis Model Assessment of insulin resistance, HOMA and the Quantitative Insulin sensitivity Check Index, QUICKI), and RA disease characteristics before and following six months of anti-TNFα treatment. Their results were compared to matched (for age, gender, BMI, disease duration and smoking status) normal-weight patients without IR (N-IR) and obese without IR (N-IR), respectively. In total, 32 patients were assessed for this study, with 8 in each group.

**Results:**

Following six months of treatment, disease activity was significantly reduced in all groups (*P *< 0.05) to a similar extent (*P *for differences between groups > 0.05 in all cases). In the total population, changes in HOMA (mean reduction at 6 m = -0.2 ± 0.1; *P *= 0.088) and QUICKI (mean increase at 6 m = 0.03 ± 0.022; *P *= 0.092) after treatment were not statistically significant, though a trend towards improvement was observed. However, N+IR patients showed a significant decrease in HOMA (mean reduction at 6 m = -0.54 ± 0.2; *P *= 0.002) and increase in QUICKI (mean increase at 6 m = 0.046 ± 0.02; *P *= 0.011). These changes were significantly different compared to the other groups (*P *< 0.05 in all cases). Multivariable analyses showed that the change in Erythrocyte Sedimentation Rate (ESR), and the change in C-Reactive Protein (CRP) associated with the improvement in HOMA (ESR: F_1-7 _= 5.143, *P *= 0.019; CRP: F_1-7 _= 3.122, *P *= 0.022) and QUICKI (ESR: F_1-7 _= 3.814, *P *= 0.021; CRP: F_1-7 _= 2.67; *P *= 0.041) only in the N+IR group.

**Conclusions:**

Anti-TNFα therapy, through controlling inflammation, seems to improve insulin sensitivity in normal-weight RA patients with insulin resistance, but is not sufficient to achieving the same beneficial effect in obese RA patients with insulin resistance.

## Introduction

Insulin resistance (IR), is a well established risk factor for the development of cardiovascular disease (CVD) [1]. The mechanisms of IR are under intense investigation; however, a consistent finding of such research is the close association between IR and inflammation [2-4]. Tumour necrosis factor alpha (TNFα), a pro-inflammatory cytokine, is thought to be one of the main mediators of IR [2]. Patients with IR exhibit increased circulating levels of TNFα [5,6], and administration of TNFα induces IR in healthy individuals [7]. In otherwise healthy individuals, obesity is a significant contributor to IR; obesity is a low-grade inflammatory condition [8,9] and TNFα is also thought to be the link between obesity and insulin resistance [3].

Rheumatoid arthritis (RA), associates with reduced life expectancy compared to the general population [10], mainly due to increased prevalence of CVD, and increased morbidity and mortality from CVD compared to the general population [11-13]. TNFα is central to the development and progression of RA and a common therapeutic target [14]. Apart from disease activity, treatment with anti-TNFα appears to also improve insulin sensitivity [15] and to reduce CVD risk in RA [16,17]. However, obesity - a potent contributor to IR in the general population - might influence the way anti-TNFα therapy affects IR. Indeed, in the general population, anti-TNFα does not improve IR in obese individuals [18]. The aim of this longitudinal study was to compare the effects of six months of anti-TNFα therapy on IR between normal weight and obese RA patients. Our primary hypothesis was that the possible beneficial effects of anti-TNFα on IR would be limited by the presence of obesity.

## Materials and methods

### Participants

The study was conducted at the Dudley Group NHS Foundation Trust, UK. It had Research Ethics Committee approval by the Black Country Ethics Committee and local R&D approval, and all volunteers provided written informed consent. Patients with RA, who were either normal weight with IR (N+IR) or obese with IR (O+IR) and embarked, for the first time, on clinically-indicated anti-TNFα treatment were invited to participate. Type of medication was decided by their managing physician and dosage was based on NICE guidelines. Patients with diabetes mellitus or using anti-diabetic medication were excluded from the study. The results of the N+IR and O+IR patients were compared to age, gender, BMI, disease duration and smoking status matched normal-weight patients without IR (N-IR) and obese patients without IR (N-IR), respectively. A total of 32 patients were assessed; 8 in each of the groups: that is, N+IR, O+IR, N-IR and O-IR. Demographic and disease characteristics appear in Table 1.

**Table 1:** Participant characteristics at baseline assessment

	Normal Weight (*n *= 16)		Obese (*n *= 16)	
	+IR	-IR	+IR	-IR
**N (females)**	8 (5)	8 (5)	8 (6)	8 (6)
**Anti-TNFα agent (Infliximab/Etanercept/Adalimumab)**	5/3/0	4/3/1	6/2/0	5/3/0
**Smokers (current; ex)**	3 (1; 2)	4 (1; 3)	3 (2; 1)	4 (2; 2)
**Age (years)**	60.8	62.2	58.6	60.8
	(6.9)	(7.8)	(6.7)	(8.0)
**Height (cm)**	165.9	167	165.4	165.2
	(10.6)	(12.2)	(11.9)	(9.4)
**Weight (kg)**	60.3	61.6	88.9	89.8
	(6.4)	(7.2)	(10.1)	(9.6)
**BMI (kg/m^2^)**	21.8	22.1	32.3	32.8
	(2.4)	(2.2)	(3.0)	(3.1)
**HOMA**	2.9	2.2*	3.1	2.1^#^
	(0.7)	(0.4)	(0.5)	(0.8)
**QUICKI**	0.29	0.36*	0.30	0.37^#^
	(0.02)	(0.03)	(0.03)	(0.01)
**HAQ**	1.6	1.8	1.7	2.0
	(0.3)	(0.2)	(0.4)	(0.5)
**DAS**	5.7	5.9	6.2	6.1
	(0.7)	(0.5)	(1.0)	(0.6)
**ESR (mm/h)**	31	38	35	43
	(7.5)	(10)	(9.5)	(12.5)
**CRP (mg/L)**	29	32.5	31.4	34.2
	(6.4)	(8.2)	(8.8)	(7.4)
**Disease Duration**	9.1	8.6	8	10.2
**(years)**	(2.5)	(2.3)	(3.6)	(4.7)

* Significantly different compared to N+IR (*P *< 0.05). ^# ^Significantly different compared to O+IR (*P *< 0.05). BMI, body mass index; CRP, C-reactive protein; DAS, disease activity score 28; ESR, erythrocyte sedimentation rate; HAQ, Health Assessment Questionnaire; +IR, with insulin resistance; -IR, without insulin resistance; HOMA, Homeostasis Model Assessment Of Insulin Resistance; QUICKI, Quantitative Insulin Sensitivity Check Index

### Assessments

Standing height was measured to the nearest 0.5 cm on a Seca 214 Road Rod portable stadiometer (Seca gmbh & co. kg., Hamburg, Germany). Body weight was assessed on a Tanita MA-418 BC body composition analyser (Tanita Corp., Tokyo, Japan). BMI (kg/m^2^) was calculated on the basis of measured height and weight. The recently published RA-specific BMI cut-off points (that is, 23 kg/m^2 ^for overweight and 28 kg/m^2 ^for obesity) were used to classify them as under-, normal-, over-weight or obese [19].

Glucose and insulin were assessed in venous blood collected in the fasting state; IR was evaluated using the Homeostasis Model Assessment of insulin resistance (HOMA) [20] and the Quantitative Insulin sensitivity Check Index (QUICKI) [21], and was defined as HOMA ≥2.5 [22] and/or QUICKI ≤0.333 [23,24]. Both methods correlate well with insulin clamp (HOMA: r = 0.88 [22]; QUICKI: r = 0.78 [21]) and are considered valid calculations for insulin resistance and insulin sensitivity, respectively. Smoking status was also recorded.

Inflammatory load was assessed by the erythrocyte sedimentation rate (ESR) and C-reactive protein (CRP). The Disease Activity Score-28 (DAS28) was used to assess clinical disease activity [25] and the anglicised version of the Stanford Health Assessment Questionnaire (HAQ) [26] to measure functional disability. Disease duration was recorded from review of the clinical notes. All assessments were repeated following six months of anti-TNFα therapy.

### Data management and analyses

The Statistical Package for Social Sciences version 15.0 (SPSS Inc. Chicago, IL, USA) was used for the statistical analyses. The Kolmogorov-Smirnov test of normality was used to assess dispersion of the variables. Dispersion of data is reported as the mean (standard deviation-SD) as all were normally distributed. Statistical significance was set at *P *< 0.05.

T-tests were used to identify differences at baseline in the assessed variables between N+IR and N-IR as well as O+IR and O-IR. Repeated measures analysis of variance (ANOVA) was then used to evaluate the effects of six-month anti-TNFα treatment on HOMA and QUICKI as well as BMI, and RA characteristics, and identify differences between groups in the magnitude of these effects. Multivariate ANOVA (MANOVA) was then used to assess the associations between the changes in HOMA and QUICKI with baseline BMI, RA characteristics (ESR, CRP, DAS, HAQ), age and gender, as well as the change in these variables following treatment. Interactions between baseline BMI and age, disease duration and CRP were also included in the models.

## Results

At baseline, t-tests did not identify any significant differences between the groups, other than, as expected, in IR (HOMA and QUICKI; Table 1). Following the six-month intervention, repeated measures ANOVA found no changes in BMI in any of the groups (*P *> 0.05). Inflammation and disease activity were significantly reduced in all groups (*P *< 0.05) but to a similar extent (*P *for differences between groups > 0.05 in all cases). Overall, HOMA showed a tendency to reduce (*P *= 0.088) while QUICKI a tendency to increase (*P *= 0.092) (Table 2).

**Table 2:** Effects of the treatment on the assessed variables for all participants

	Δ6 m	*P*
**BMI (kg/m^2^)**	0.45 ± 0.07	0.466
**DAS**	-2.37 ± 0.4	0.000
**HAQ**	-0.4 ± 0.01	0.001
**ESR (mm/h)**	-18.55 ± 12.8	0.002
**CRP (μg/L)**	-16.1 ± 9.4	0.016
**HOMA**	-0.2 ± 0.1	0.088
**QUICKI**	0.03 ± 0.022	0.092

Δ6 m, mean difference between baseline and six-month measurement; BMI, body mass index; CRP, C-reactive protein; DAS, Disease Activity Score 28; ESR, erythrocyte sedimentation rate; HAQ, Health Assessment Questionnaire; HOMA, Homeostasis Model Assessment Of Insulin Resistance; QUICKI, Quantitative Insulin Sensitivity Check Index.

**Figure 1:**
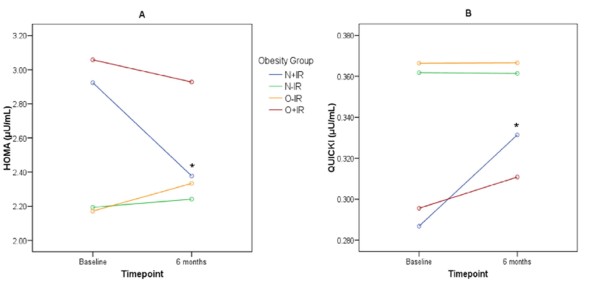
**Changes in HOMA and QUICKI following six months of anti-TNFα treatment**. Anti-TNFα treatment significantly improves HOMA and QUICKI only in the N+IR group. * Significant difference compared to baseline. HOMA, homeostasis model assessment of insulin resistance; QUICKI, quantitative insulin sensitivity check index; N+IR, normal weight with insulin resistance; N-IR, normal weight without insulin resistance; O+IR, obese with insulin resistance; O-IR, obese without insulin resistance.

When patient grouping was introduced as a factor in the analyses, it was revealed that the treatment resulted in significant decreases in HOMA (Δ6 m = -0.54; *P *= 0.002) and increases in QUICKI (Δ6 m = 0.046; *P *= 0.011) in the N+IR, but not any of the other three groups (*P *> 0.05 in all cases). The magnitude of the changes in IR was significantly different between N+IR and the rest of the groups (N-IR: HOMA, *P *= 0.008, QUICKI, *P *= 0.002; O+IR: HOMA, *P *= 0.019, QUICKI, *P *= 0.038; O-IR: HOMA, *P *= 0.000, QUICKI, *P *= 0.001; Figure 1). No differences in the magnitude of improvements between the other groups were observed (*P *> 0.05 for both HOMA and QUICKI).

Finally, MANOVA indicated that baseline BMI, baseline RA characteristics, baseline BMI, and the change in BMI did not associate with the change in HOMA or QUICKI in any of the groups (*P *> 0.05 in all cases). However, the change in ESR and the change in CRP associated with the improvement in HOMA (ESR: F_1-7 _= 5.143, *P *= 0.019; CRP: F_1-7 _= 3.122, *P *= 0.022) and QUICKI (ESR: F_1-7 _= 3.814, *P *= 0.021; CRP: F_1-7 _= 2.67; *P *= 0.041) only in the N+IR group.

Retrospective power calculations (a = 0.05) using the values observed above indicate a power of 0.91 and a power of 0.88 for the changes observed in HOMA (Δ6 m for N+IR = 0.49, for O+IR = 0.11, common St.D. = 0.26) and QUICKI (Δ6 m for N+IR = 0.04, for O+IR = 0.01, common St.D. = 0.02), respectively.

## Discussion

The main aim of this study was to compare the effects of anti-TNFα treatment on insulin sensitivity among normal weight and obese RA patients. To our knowledge, this is the first study to use such an approach and indeed to identify different effects of anti-TNFα treatment in normal-weight vs. obese individuals with RA. Our results indicate a significant improvement of IR only in normal-weight RA patients with IR but not in obese RA patients with IR. Reduction in inflammation was associated with the changes in IR only in the normal weight but not in obese patients.

Sample size is the main limitation of our study. However, the number of patients that fulfilled the inclusion criteria for this study (that is, insulin resistance, anti-TNFα naive and embarking on anti-TNFα) is limited. This is also reflected by sample size of other studies investigating similar hypotheses [15]. In our study, a larger sample size might have been able to identify improvements in IR in the O+IR group. This group had a slight improvement in IR (as shown in Figure 1). However, this improvement was not statistically significant (mean change in HOMA: -0.11; 95% odds ratio: 0.06 to 0.15; and QUICKI: 0.009; 95% odds ratio: 0.004 to 0.018; *P *> 0.05 for both) or indeed clinically significant as both variables remained within the "at risk" range. Moreover, the statistical test we used (Repeated measures ANOVA) looks at the differences in the responses between the two groups, not merely the response of each group. We, therefore, doubt that a larger sample size would significantly change the conclusions of the present study. A second limitation of our study is that we did not control for changes in physical activity, which may influence IR. However, any such changes are likely to have occurred throughout all the subgroups, especially since disease activity was reduced equally among them. Additionally, for the duration of the study, no changes in thyroid status were observed (all patients were euthyroid at baseline), there were no changes in other anti-rheumatic medication (including steroids and hydroxychloroquine) or cardio/vasoactive therapy (including angiotensin-converting-enzyme (ACE) inhibitors).

Similar studies in RA patients have shown reduction in IR following anti-TNFα therapy (reviewed in [27]). BMI, even though assessed, is not reported in some of these studies [28,29]; in those who report it, observations are predominantly made on normal-weight to slightly overweight patients (BMI: approximately 22 kg/m^2 ^[30] to approximately 25 kg/m^2 ^[31,32]). Even in these studies increasing BMI appears to associate with a smaller improvement in IR [31]. Similarly, acute administration of anti-TNFα significantly improved insulin sensitivity in most RA patients; however, this improvement was again minimised with increasing BMI [33]. We have been able to find a single case-study of an obese patient with RA that experienced significantly improved IR following anti-TNFα treatment [34]; the extremely high baseline HOMA levels (> 25 with a cut-off for IR at 2.5) of this patient should be noted.

The reason for this apparent difference in the responses of lean vs. obese individuals to anti-TNFα is not yet clear. However, our findings for the obese RA patients are in line with data from the general population. In obese individuals with the metabolic syndrome [18] or with type 2 diabetes mellitus [35] anti-TNFα failed to improve IR. It is well established that enlarged adipose tissue is a source of inflammation. Up to 50% of its cell mass is monocytes and macrophages. Adipocytes and macrophages both are a source of inflammatory cytokines reducing insulin sensitivity of muscle and increasing plasma concentrations of these compounds [36,37]. The cytokines TNFα and interleukin-6 (IL-6) in particular, stimulate both the c-Jun amino-terminal kinase (JNK) and the IκB kinase-β (IKK-β)/nuclear factor-κB (NF-κB) pathways, resulting in up-regulation of potential mediators of inflammation and lead to IR [38]. Other potential pathways by which obesity might induce IR include the action of retinol-binding protein-4 (RBP4) which reduces phosphatidylinositol-3-OH kinase (PI(3)K) signalling in muscle and enhances expression of the gluconeogenic enzyme phosphoenolpyruvate carboxykinase in the liver [39]. The role of IL-6 is of particular importance in the context of the present study, as this is not directly targeted by anti-TNFα medication and could thus be important in maintaining IR in obese individuals. A final interesting approach to the potential underlying mechanisms could be the role of the inflammasome and, especially, NLRP3 in IR. This is a cytosolic protein complex leading to the activation of the processing enzyme caspase-1, which is central to the production of Interleukin -1β (IL-1β) [40]. IL-1β has been recently recognized as a potent instigator of the obesity-induced inflammation and as such a contributor to IR [41]. Even though anti-TNFα medication does not directly target IL-1β, it is implicated in the activation of NLRP3 [42], thus indirectly affecting IL-1β production. The associations of obesity, inflammasomes and IL with IR in RA warrant further investigation.

Adipose tissue also secretes adipokines, such as adiponectin and resistin [43], reduced levels of adiponectin and increased levels of resistin associate with IR [44,45]. IR and other features of the metabolic syndrome independently associate with atherosclerosis in RA [46]. Adiponectin stimulates fatty acid oxidation via AMP-activated protein kinase (AMPK) and peroxisome proliferator activated receptor-α-dependent pathways [38].

Anti-TNFα treatment may affect levels of these adipokines. In patients with severe RA, refractory to conventional disease-modifying antirheumatic drug (DMARD) therapy, periodical treatment with infliximab (an anti-TNFα blocker) results in a rapid reduction of serum resistin levels [47]. Serum resistin levels, in these patients, associate with laboratory markers of inflammation, particularly CRP, but not with BMI [47]. Moreover, in the same cohort of patients, an independent negative correlation of high-grade inflammation with circulating adiponectin concentrations has been observed [48]. Low adiponectin concentrations further correlate with atherogenic dyslipidemia and high plasma glucose [48].

Moreover, anti-TNFα treatment may reduce lipolysis within the muscle by inhibiting TNFα action locally [43] - obese individuals have high fat intramuscular content. This may lead to further increases in intramuscular concentration of fatty acid metabolites, such as diacylglycerol, fatty acyl-coenzyme A, and ceramides, as a result of increased release of non-esterified fatty acids [39]. This leads to serine/threonine phosphorylation of insulin receptor substrate-1 and -2, and eventually to insulin resistance [49].

From a clinical point of view, our findings would suggest that additional measures to anti-TNFα therapy should be employed in order to combat IR in obese RA patients. Weight-loss is probably the primary such measure. However, due to the lack of weight-loss studies in RA [50] and the high prevalence of muscle wasting among these patients [51], any such interventions should be employed with great care. Exercise, which is known to benefit RA patients in several ways [52], could possibly prove a valuable tool in reducing body weight while maintaining muscle mass and improving insulin sensitivity, but further research is needed. In addition to IR, such interventions could improve other CVD risk factors and reduce the high CVD risk of RA patients.

From the above results, it seems that the effects of anti-TNFα therapy on IR differ significantly between normal-weight and obese patients with RA. Such therapy seems to improve IR in normal-weight but not in the obese RA patients. In the latter group, obesity-related processes seem to counteract the potential benefits of anti-TNFα. Further studies looking at the specific mechanisms by which anti-TNFα therapy affects IR and the mechanisms by which obesity interacts in this process should be pursued.

## Conclusions

In conclusion, anti-TNFα therapy seems to improve insulin sensitivity in normal weight RA patients with IR but not in obese RA patients with IR. It is possible that the mechanisms leading to insulin resistance are partly different in normal-weight and obese patients with RA.

## Abbreviations

ACE inhibitor: angiotensin-converting-enzyme inhibitor; AMPK: AMP-activated protein kinase; ANOVA: analysis of variance; BMI: body mass index; CRP: C-reactive protein; CVD: cardiovascular disease; DAS28: Disease Activity Score-28; DMARD: disease-modifying antirheumatic drug; ESR: erythrocyte sedimentation rate; HAQ: Health Assessment Questionnaire; HOMA: Homeostasis Model Assessment of Insulin Resistance; IKK-β: IκB kinase-β; IL-6: interleukin 6; IR: insulin resistance; +IR: normal-weight with insulin resistance; JNK: c-Jun amino-terminal kinase; MANOVA: multivariate analysis of variance; NF-κB: nuclear factor-κB; N-IR: normal-weight without insulin resistance; NO+IR: obese with insulin resistance; O-IR: obese without insulin resistance; PI(3)K: phosphatidylinositol-3-OH kinase; QUICKI: Quantitative Insulin sensitivity Check Index; RA: rheumatoid arthritis; RBP4: retinol-binding protein-4; TNFα: tumor necrosis factor alpha.

## Competing interests

The authors declare that they have no competing interests.

## Authors' contributions

AS was involved in the conception and design of the study, and in data collection, data analyses and the writing of the manuscript. GM participated in the data collection and manuscript writing. VP was involved in data collection and data analyses. PN is the statistics expert of the team and led data analyses. YK was involved in the design of the study and drafting of the manuscript. GK was involved in the conception and design of the study, drafting of the manuscript, and acted as the study supervisor. All authors have read and approved the manuscript for publication.
